# Theophylline-Ethylcellulose Microparticles: Screening of the Process and Formulation Variables for Preparation of Sustained Release Particles

**Published:** 2012

**Authors:** Mitra Jelvehgari, Siavoush Dastmalch, Derafshi Nazila

**Affiliations:** 1 *Department of Pharmaceutics, Faculty of Pharmacy, Tabriz University of Medical Sciences, Tabriz, Iran*; 2 *Department of Medicinal Chemistry, Faculty of Pharmacy, Tabriz University of Medical Sciences, Tabriz, Iran*; 3 *Biotechnology Center, Tabriz University of Medical Sciences, Tabriz, Iran*

**Keywords:** Emulsion Solvent Diffusion (ESD), Ethylcellulose, Release, Theophylline.

## Abstract

**Objective(s):**

The aim of this study was to formulate and evaluate microencapsulated controlled release preparations of theophylline using ethylcellulose as the retardant material with high entrapment efficiency.

**Materials and Methods:**

Microspheres were prepared by water-in-oil-in-oil (W/O_1_/O_2_) emulsion-solvent diffusion (ESD). A mixed solvent system consisting of acetonitrile and dichloromethane in a 1:1 ratio and light liquid paraffin were chosen as primary and secondary oil phases, respectively. In the current study formulations with different drug/polymer ratios were prepared and characterized by drug loading, loading efficiency, scanning electron microscopy (SEM), X-ray diffraction (XRD), Fourier Transform infrared spectroscopy (FTIR) and differential scanning calorimetry (DSC).

**Results:**

The best drug to polymer ratio was 0.5:1 (F_2_ formulation). F_2_ Formulation showed 29.53% of entrapment, loading efficiency of 88.59%, and mean particle size of 757.01 µm. SEM studies showed that the microspheres were spherical. FTIR, SEM, XRD and DSC showed that drug in the microspheres was stable and revealed crystallinity form.

**Conclusion:**

The results showed that, generally, an increase in the ratio of drug to polymer resulted in a reduction in the release rate of the drug which may be attributed to the hydrophobic nature of the polymer. The release of theophylline was found to be diffusion controlled and was influenced by the drug to polymer ratio, loading efficiency, and particle size. The *in vitro* release profile could be modified by changing various processing and formulation parameters (as stirring rate, the volume of dispersing medium, and non-solvent concentration) to give a controlled release of drug from the microparticules.

## Introduction

Xanthine derivatives, theophylline (TH) is considered to be respiratory smooth relaxants, they also have other pharmacologic actions. They are used both as a prophylactic drug and as a means to prevent acute exacerbations of asthma. The short plasma half-life (4-8 hours) following oral dosing (1) necessitates frequent administration of the drug in order to maintain the desired steady state levels. Patient compliance is also known to be fairly poor with such frequent dosing regimens (2). Furthermore, dosage forms exhibiting slow release have been developed (3-5). Thus, clinical benefit and related advantages are likely to happen if such a drug were to be administered as a modified release dosage form. A multiple-unit system was proposed in view of the many advantages that these dosage forms offer (6). Ethylcellulose (EC) was chosen as a matrix polymer for preparation of microcapsules showing prolonged release of theophylline. 

Microencapsulation has been used as one of the methods to deliver drugs in a controlled manner (7). The selection of a particular encapsulation method is primarily determined by the solubility characteristics of the drug and polymer (8). A popular method for the encapsulation of water-soluble drugs within water insoluble polymers is the double-emulsion solvent diffusion method (9). One method for ensuring high entrapment efficiency of water soluble active ingredients is to use a hydrophobic processing medium out of which the hydrophilic drug molecule is unlikely to migrate. The encapsulation of TH in cellulose acetate butyrate and Eudragit Rl-100, cellulose acetate butyrate and cellulose acetate phthalate, ethyl cellulose microcapsules has been described in previous works (10-12). Moreover, ethylcellulose theophylline loaded microcapsules prepared by the O/W emulsion non-solvent addition method may show the slower release rate than the free drug (Tsai 2001). However, most of the microencapsulation techniques have been used for lipophilic drugs, since hydrophilic drugs showed low loading efficiency (13). 

Bagory investigated the effects of different ethylcellulose: theophylline ratios and different plasticizer (diethylphthalate) concentrations on theopylline release (14). Proper dissolution profile was observed for microspheres with 1:1 ethylcellulose to theophylline ratio, with 600-l000 m particle size, and 20% plasticizer (14). Encapsulation using oil as the processing medium was chosen with the expectation that TH, the hydrophilic drug, would find it unfavourable to diffuse out of the microspheres before hardening. Dashevsky et al, showed the effect of ethylcellulose molecular weight on properties of theophylline microcapsules by using W/O emulsion-solvent evaporation method (16). The size distribution of microspheres was dependent on the ratio of ethylcellulose mixtures with high and low molecular weights (15).

Sudip *et al* reported that theophylline was entrapped in ethyl cellulose microcapsules by a water/oil/water emulsification-solvent evaporation method (16). Due to its hydrophilicity, TH is likely to preferentially partition out into the aqueous medium, leading to low entrapment efficiency, when encapsulated using the aqueous phase as the processing medium (16). The release profile was significantly affected by added polyisobutylene and varying concentration of it. A further effect of partitioning is the accumulation of drug crystals on the surface of microspheres which produces burst release of the drug on administration (16). The double emulsion technique has fairly good encapsulation efficiency for hydrophilic compounds; however, particle size is usually larger with single emulsion technique (16). 

In the present investigation, TH was incorporated in ethylcellulose microparticles by using W/O_1_/O_2_ diffusion-solvent emulsion method, with the aim of improving loading efficiency. This work undertakes to determine the influence of formulation variables (drug-polymer ratio, stirring rate, dispersing medium and emulsifier concentrations) on *in vitro *drug release and micromeritic properties (drug content, incorporation efficiency, yield value, particle size diameter, size distribution, and surface characteristics) of the prepared microspheres*.*

## Material and Methods

*Materials*

Theophylline was provided from Merck, Germany, ethyl cellulose 48 cP from Sigma-Aldrich, USA, and Theophylline SR^®^ from Daru pakhsh, Iran, dichloromethane, acetonitrile, span 80, Liquid paraffin, n- hexane, hydrochloric acid, potassium phosphate dibasic, sodium hydroxide (Merck, Germany). All solvents and reagents were of analytical grade.

***Experimental Methods***

***Preparation of microcapsules***

Microcapsules were prepared by water-in oil-in oil (W/O_1_/O_2_ double emulsion solvent diffusion method) using different ratios of TH to ethylcellulose (0.25:1, 0.5: 1, 0.75: 1 and 1:1 as shown in Table 1), as described in the previous study (17). In the first step, an aqueous solution of drug (150 mg/2 ml) used as the internal aqueous phase was emulsified into an organic solution of the polymer (300 mg ethyl cellulose) was dissolved in 5 ml of the mixed solvent system consisting of acetonitrile and dichloromethane in a 1:1 ratio. The initial W/O emulsion was prepared by adding 2 ml of aqueous phase to the polymer solution while stirring using a mechanical stirrer at 500 rpm. This W/O primary emulsion was slowly added to 50 ml of light liquid paraffin, the second oil phase containing 0.5% span 80 as a surfactant while stirring by a paddle propeller at 1000 rpm, immersed in an ice water bath. After 2 hrs, 10 ml of n-hexane (non-solvent) was added to harden the microcapsules and stirring was continued for a further 1 hr and the hardened microcapsules were collected by filtration and washed with three portions of 50 ml of n-hexane and were air dried for 12 hrs (17). 

***Determination of drug content of microcapsules***

The total drug loadings were determined by dissolving 20 mg TH microcapsules of each sample in 10 ml dichloromethane and measuring the absorbance at 274.8 nm using a spectrophotometer. All experiments were done in triplicate.

***Determination of drug entrapment efficiency and production yield***

The loading efficiency (%) was calculated according to the following equation:

Drug entrapment efficiency (%) = (Experimental drug content/initial drug content in the formulation) × 100

The yield of the process was expressed as a percentage of the dried microparticles with respect to the starting mass of the TH and the polymer used. All of the experiments were performed in triplicate and the mean of the values was reported (17).

***Viscosity measurement***

A Brookfield rotational digital viscometer DVLV-II was used to measure the viscosity (cP) of the internal and external phases at 25 ^◦^C. Spindle numbers 1 was rotated at 100 rpm.

**Table 1:** Theophylline microparticle formulations prepared by Double-Emulsion Solvent Diffusion Method (w/o_1_/o_2_)

Formulations	Drug: Polymer ratio	Initial emulsion (W/O_1_)					Secondary phase oily (O_2_)	
		Aqueous Phase (W)		Initial organic phase (O_1_)			Liquid paraffin (ml)	Span 80 (%)
		water (ml)	theophylline (mg)	ethylcellulose (mg)	acetonitrile (ml)	dichloromethane (ml)		
F_1_ F_2_ F_3_ F_4_	0.25:1 0.5:1 0.75:1 1:1	2 2 2 2	75 150 225 300	300 300 300 300	2.5 2.5 2.5 2.5	2.5 2.5 2.5 2.5	50 50 50 50	0.5 0.5 0.5 0.5

***Differential scanning calorimetry (DSC)***

DSC thermogram of the pure drug and microcapsules were recorded with differential scanning calorimetery (DSC 60, ). Typically, about 5 mg of sample was weighed into an aluminum pan, the pan crimped non-hermetically, and was heated in the differential scanning calorimeter from 30 to 300 ˚C at a rate of 10 ˚C per min.

***X-ray powder diffractometry (X-RPD)***

X-ray diffraction spectrum of the pure drug and microcapsules were recorded with a (Siemens D5000, ) using nickel-filtered CuKα radiation (a voltage of 40 KV and a current of 20 mA). The scanning rate was 2˚/min over a 2θ range of 20-60˚ and with an interval of 0.02 ˚C.

***Fourier-Transform infrared spectroscopy (FTIR)***

The infrared spectra of the pure drug and microcapsules were recorded using a FTIR (Bomen ) spectrophotometer between the ranges 500 to 4000 cm^-1^ by making a pellet of the samples with potassium bromide discs (0.5% w/w).

***Scanning electron microscopy (SEM)***

Scanning electron microscopy (LEO 440i, England) was done to characterize surface topography of microcapsules operating at 15-kV. The samples were mounted on a metal stub with double adhesive tape and coated with platinum/palladium alloy under vacuum. 

***Particle size analysis***

A laser light scattering particle size analyzer (SALD-2101, ) was used to determine the particle size of the drug and microparticulate formulations. Samples were suspended in distilled water (Microparticles) or dichloromethane (TH) in a 1 cm cuvette and stirred continuously during the particle size analysis. 

***In vitro release study***

TH dissolution patterns from microcapsules were obtained under sink conditions. USP rotating basket method was used for all microsphere formulations. A set amount of microcapsules (200 mg drug) was added to 900 ml dissolution medium (pH 1.2 HCl solution), preheated and maintained at 37 ± 1˚C in a water bath, then stirred at 100 rate/min. Aliquots (5ml) of the solution were withdrawn at pre-set times through a 0.45 µm filter and replaced with an equal volume of fresh test fluid to keep the volume constant. After 2 hr, 17 ml of 0.2 M phosphate buffer stock, pre-equilibrated at 37 ˚C, were added to the dissolution vessel. The pH was immediately adjusted, if necessary, with 0.2 N HCl or 0.2 N NaOH to pH 7.4. Samples were suitably diluted with the same fluid, and drug concentration was measured by UV analysis at 207.6 nm (for acidic medium) or 208.6 nm (for alkaline buffer). Each experiment was performed in duplicate and a close reproducibility was attained. 

***Release characteristics and kinetics studies***

In order to have a better comparison between different formulations dissolution efficiency (DE), t_50_% (dissolution time for 50% fractions of drug); and difference factor, F_1_ (used to compare multipoint dissolution profiles) was calculated. The results are listed in Table 3 (18). Dissolution efficiency (DE) (19), defined as the area under the dissolution curve up to a certain time *t*, which is expressed as a percentage of the area of the rectangle arising from 100% dissolution in the same time. The areas under the curve (AUC) were calculated for each dissolution profile by the trapezoidal rule. DE can be calculated as follows:

$\mathrm{DE}=\int y\frac{\mathrm{dt}}{100t}$

Where *y *is the drug percent dissolved at time *t*. In this paper, all dissolution efficiencies were obtained with *t *equal to 1440 min. The in* vitro* release profiles of different microsphere formulations were compared with commercial theophylline extended release formulation using difference factor (f_1_), as defined by the following formula equation (19):

f_1_= {[Σ _t=1_^n^ |R_t_-T_t_|] / [Σ _t=1_^n ^R_t_]} ×100

Where *n *is the number of time points at which % dissolved was determined, *R*_t _is the % dissolved of one formulation at a given time point and *T*_t _is the % dissolved of the formulation to be compared at the same time point. The difference factor fits the result between 0 and 15, when the test and reference profiles are identical and approaches above 15 as the dissimilarity increases. 

Data obtained from *in vitro *release studies were fitted to various kinetic equations to find out the mechanism of drug release from the ethylcellulose microcapsules. The kinetic models used were:

Q_t_ = k_0_t (zero-order equation)

ln Q_t_ = ln Q_0_ – k_1_.t (first-order equation)

Q_t_ = K. S.√t = k_H_. √t (Higuchi equation based on Fickian diffusion)

Where, Q is the amount of drug release in time *t*, Q_0_ is the initial amount of drug in the microcapsules, S is the surface area of the microparticle and k_0_, k_1_ and k_H_ are rate constant of zero order, first order and Higuchi rate equation, respectively. In addition to these basic release models, there are several other models as well. One of them is Peppas and Korsemeyer equation (power law).

Mt/M_∞_= k.t^n^

Where M_t_ is the amount of drug release at time *t* and M_∞_ is the amount release at time *t* = ∞, thus Mt/M_∞_ is the fraction of drug released at time *t*, k is the kinetic constant, and *n* is the diffusion exponent which can be used to characterize both mechanism for both solvent penetration and drug release. Determination of the correlation coefficient assessed fitness of the data into various kinetic models. The rate constants, for respective models were also calculated from slope (15, 16). 

***Optimization of the double-emulsion solvent diffusion technique***

*Effect of stirring rate*

Various batches of the selected formulation (F_2_) were made, but the stirring rate was the only parameter that varied between 500 and 1500 rpm. After drying, the weighed batch of microcapsules was subjected to drug content, loading efficiency, particle size and drug release tests. 

*Influence of formulation variables*

The influence of formulation variables on microcapsule formation, micromeritic and drug release characteristics was investigated on formulation F_2_. These variables included the volume of dispersing medium and non-solvent concentration

## Results

***The effect of drug: polymer ratio on the physical properties of the microparticles***

**Figure 1:**
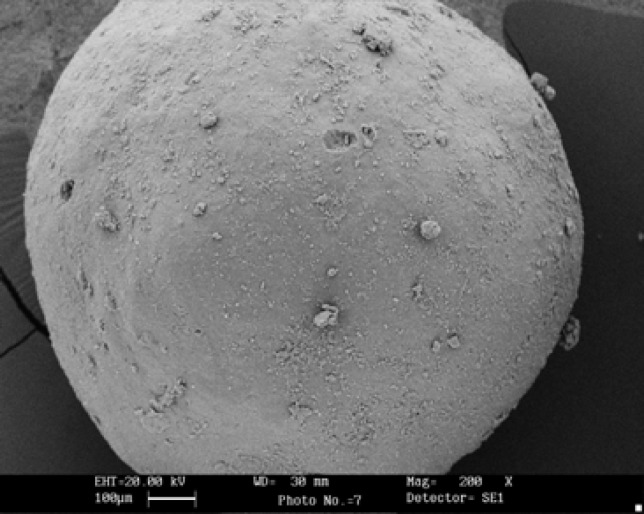
SEM of a spherical microcapsules containing theophylline F_2_ (drug: polymer ratio 0.5:1), with 200X

The results of the effect of drug-polymer ratio on production yield, drug loading efficiency and mean particle size are shown in Table 2. In all of the formulations, the mean amount of drug entrapped in prepared microcapsules ranged from 88.59 to 93.60% of the theoretical content (Table 2). Generally, increasing the drug-polymer ratio increased the production yield (55.24 to 70.25%), when the ratio of drug–polymer increased from 0.25:1 to 1:1 the production yield increased (*P*< 0.05). Mean particle size of original theophylline and ethylcellulose was 43.63±1.73 μm and 76.09±0.33 μm, respectively (Table 2). The developed microparticles were found to be discrete, spherical, free flowing and white in color. SEM was performed on the developed microcapsules to assess their surface and morphological characteristics as shown in Figure 1 (F_2_ formulation). The results of particle size analyzer showed that all formulations indicated a log probability distribution (graph not shown here) and the mean particle size of microcapsules increased from 757.01±2.72 to 829.48±7.73 µm with increasing the amount of drug (Table 2).

**Table 2:** Effect of drug: polymer ratio, stirring rate, dispersing medium and non-solvent on the content, production yield and particle size of theophylline microparticles

parameter	Characteristics						
	Formulation code	Process variable	Production yield (%±SD)	Theoreticaldrug content (%)	Mean drug entrapped (%)	Drug loading efficiency (%±SD)	Mean particle size (µm ± SD)
TOL: EC ratio	F_1_ *F_2_ F_3_ F_4_	0.25:1 0.5:1 0.75:1 1:1	- 55.24±1.19 66.19±2.44 70.25±5.17	- 33.33 42.86 50	- 29.53±4.92 38.85±0.11 46.80±0.36	- 88.59±6.31 90.64±1.32 93.60±3.15	- 757.01±2.72 781.67±5.75 829.48±7.73
Stirring rate (rpm)	F_2_-1 *F_2_-2 F_2_-3	500 1000 1500	1.38±60.35 1.19±55.24 6.31 ±44.38	33.33 33.33 33.33	2.21 ±31.38 4.92±29.53 1.04±12.23	7.18± 94.15 6.31±88.59 0.17±36.69	984.61±2.24 757.01±5.72 394.11±5.67
Volume of dispersing medium (ml)	F_2_-4 *F_2_-5 F_2_-6	25 50 100	- 1.19±55.24 4.15±38.17	- 33.33 33.33	- 4.92 ±29.53 0.24 ±11.96	- 6.31±88.59 35.88±1.90	**-** 757.01±5.72 588.76±1.73
Non-solvent concentration (%)	F_2_-7 *F_2_-8 F_2_-9	5 10 20	1.87 ±39.65 1.19 ±55.24 2.32±59.19	33.33 33.33 33.33	3.56± 32.04 4.92 ± 29.53 17.06 ±21.03	7.05 ±96.13 6.31±88.59 2.33±63.10	870.55±3.62 757.01±5.72 599.283±1.72

*F_2_ is same F_2_-2, F_2_-5 and F_2_-8 .

**Table 3:** Comparison of various release characteristics of theophylline from different microsphere formulations, physical mixture and theophylline SR^® ^Tablet

Formulation code	Rel_ 2_^a^ (%)	^b^ Rel _8_ (%)	DE ^c^	^d^ t _50%_ (h)	^e^ f_1_
F_2_	25±2.10	91.87±3.40	80.48±4.21	4	32.59±2.23
F_3_	41.49±1.26	95.13±0.43	82.83±2.11	3	46.31±3.67
F_4_	46.26±2.23	96.72±2.23	85.36±3.55	3	54.28±4.42
Physical mixture	77.83±0.35	77.83±0.35	103.79±5.52	-	120.59±6.34
Theophylline SR^®^	12.89±1.55	80.86±5.73	73.72±3.98	4	0

amount of drug release after 2 hr^b^amount of drug release after 8 hr,^ c^Dissolution Efficiency,^ d^dissolution time for 50% fractions,^ e^difference factor.

The results showed that the apparent viscosities of the different drug polymer ratios (0.25:1, 0.5:1, 0.75:1, 1:10) were 13, 21, 30 and 40.2 mPa.S respectively**.** When the viscosity of the ispersed phase of these formulations was investigated, it was found that particle sizes of microparticles were directly proportional to the apparent viscosity of dispersed phase. Higher actual drug loading were obtained by increasing the theoretical drug loading (1). In all cases, the encapsulation efficiencies were similar and greater than 80%.

***Scanning electron microscopy (SEM)***

As shown in SEM photographs (Figure 1) microcapsules were spherical.

**Table 4:** Fitting parameters of the *in vitro* release data to various release kinetics models

Order		F2	F3	F4	Theophylline SR
Zero (f=kt)	0.0006	0.0005	0.00005	0.0007	K
	0.5148	0.5249	0.5327	0.6869	RSQ
	723.1258	784.3812	808.0542	459.3552	D(SS)%
First (Ln(1-f)= kt)	0.0022	0.0023	0.0025	0.0032	k
	0.7205	0.7204	0.7333	0.9936	RSQ
	309.546	406.6294	402.1296	745.9072	D(SS) %
Peppas (Lnf=lnk+blnt)	0.34091	0.3960	0.2805	1.2330	b
	0.0518	0.064	0.1167	0.0004	k
	0.9433	0.9558	0.9736	0.9725	RSQ
	22.4856	26.1420	425.3395	97.9302	D(SS) %
Higuchi (f=kt ^0.5^)	0.0295	0.0262	0.0243	0.0339	k
	0.7260	0.7509	0.7551	0.8721	RSQ
	201.3116	332.3816	425.3395	1398.717	D(SS) %

***Differential scanning calorimetry (DSC)***

**Figure 2:**
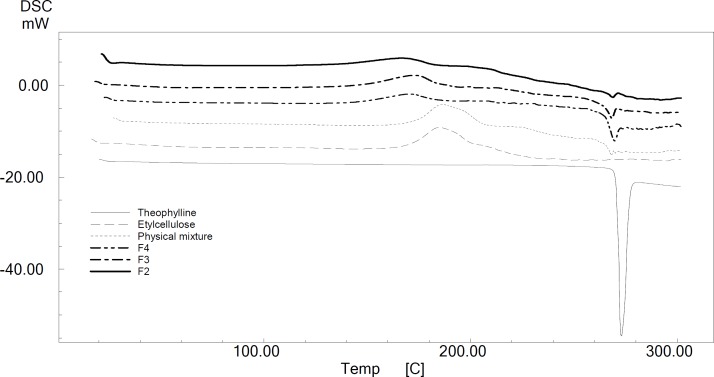
DSC thermogram of theophylline, ethylcellulose and microsphere formulations

DSC of TH showed a sharp endothermic peak at 271.41 °C, as given in Figure 2. TH in the ethylcellulose microcapsules also showed a similar characteristic peak (Figure 2) with varying intensity. 

***X-ray powder diffractometry***

**Figure 3:**
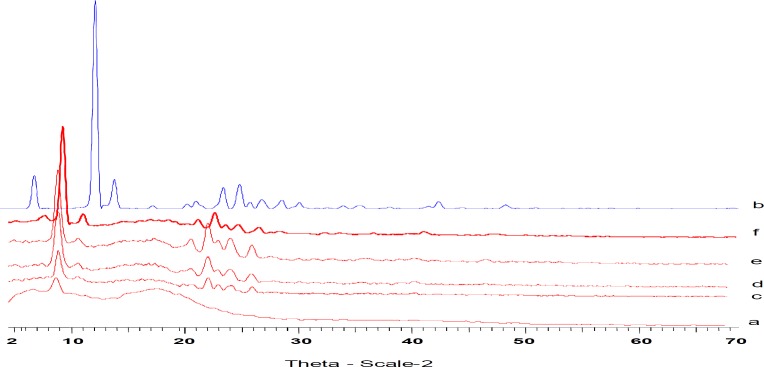
X-ray diffraction of ethylcellulose (a); theophylline (b); Physical Mixture (c), F_2_ (d); F_3_ (e), and F_4 _(f)

Figure 3 illustrates the comparative X-ray powder diffraction pattern of TH alone, physical mixture of TH with ethylcellulose and TH-loaded ethylcellulose microcapsules.

***Fourier transform infrared spectroscopy (FTIR)***

**Figure 4:**
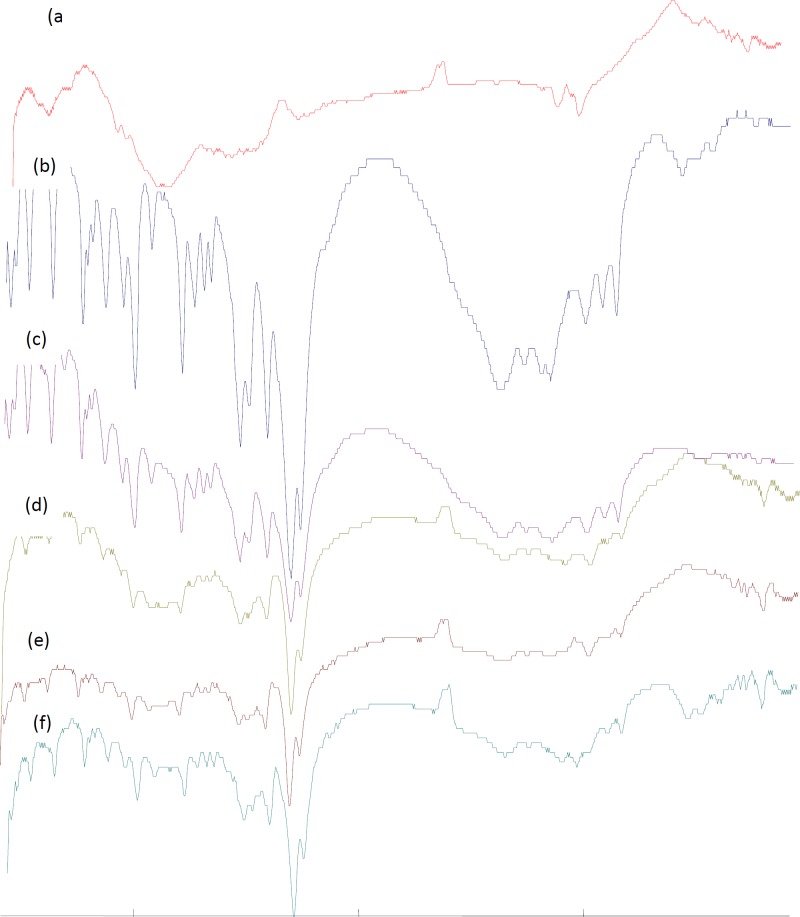
FTIR spectrum of ethylcellulose (a); theophylline (b); Physical Mixture (c), F_2_ (d); F_3_ (e), and F_4 _(f)

The infrared spectrum of TH and TH-loaded microcapsules was identical and the loaded microsphere is of lower intensity than the drug alone, as shown in Figure 4.

***Effect of stirring rate***

The effect of stirring rate on the physical characteristics of the microcapsules is shown for formulation F_2_. The results of stirring rate on the mean particle diameter of microcapsules, drug entrapment and production yield are listed in Table 2. The results showed that increasing the stirring rate from 500-1500 rpm decreased the production yield and the drug content (*P*< 0.05).

***Effect of volume of dispersing medium***

The volume of processing medium (outer phase, O_2_) significantly influenced the entrapment efficiency of the microcapsules (Table 2). When 25 ml n-hexane was incorporated, microcapsules were not formed because the low non-solvent content failed to prevent droplet coalescence in the oil medium; as a result mean particle size increased. As the volume of processing medium increased from 25 ml to 100 ml, the entrapment efficiency significantly decreased from 88.60% to 35.88% (comparing F_2_-5 and F_2_-6) (*P*< 0.05).

***Effect of non-solvent concentration***

When the concentration of non-solvent increased, production yield increased (*P*< 0.05), whereas drug content and the mean particle size of microcapsules decreased (comparing formulations F_2_-7, F_2_-8 and F_2_-9 in Table 2). According to Table 2, when non-solvent concentration increased, size of microparticles in F_2_-8 and F_2_-9 (respectively 10 and 20 ml non-solvent) was smaller than F_2_-7. 

***In vitro release studies***

**Figure 5:**
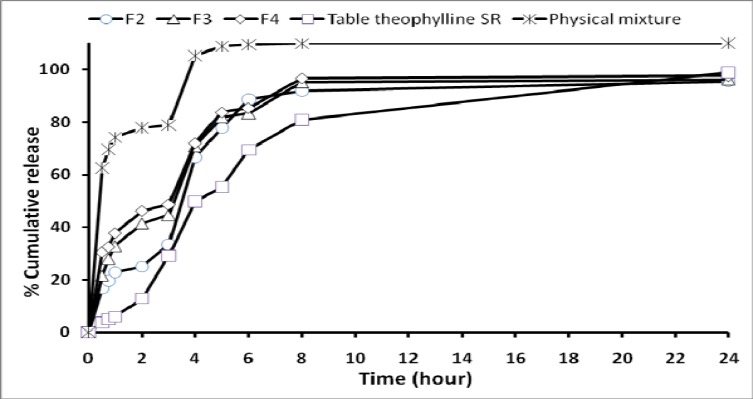
Cumulative percent release of theophylline from microspheres prepared with different polymer-to-drug ratios, physical mixture and theophylline SR® tablet

Figure5 illustrates the *in vitro* release profile of each formulation, in pH 1.2 and 7.4, by representing the percentage of TH release with respect to the amount of TH encapsulated. However, with respect to the physical mixture, microparticles showed faster release of drug in pH 7.4. For microparticles, dissolution of TH at pH 1.2 was strongly reduced and the initial burst effect at pH 7.4 was moderated, resulting in an overall slower drug release.

However, Figure 5 shows that the burst effect was higher when the TH to polymer ratio was 1:1 (F_4_) and 0.75:1 (F_3_). Therefore F_4_ and F_3_ formulations could not prolong the release of TH.

**Figure 6:**
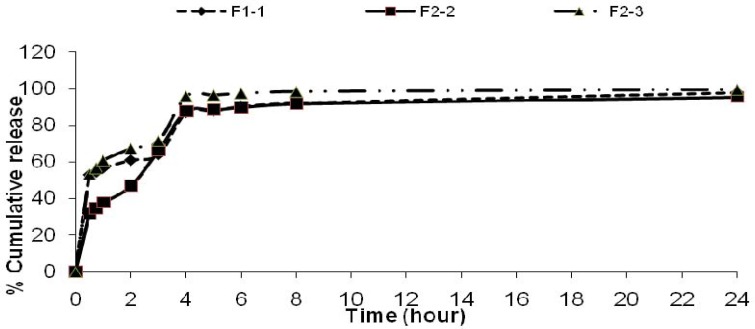
Cumulative percent release of theophylline from microspheres F_2_ prepared with different rates of stirring

**Figure 7:**
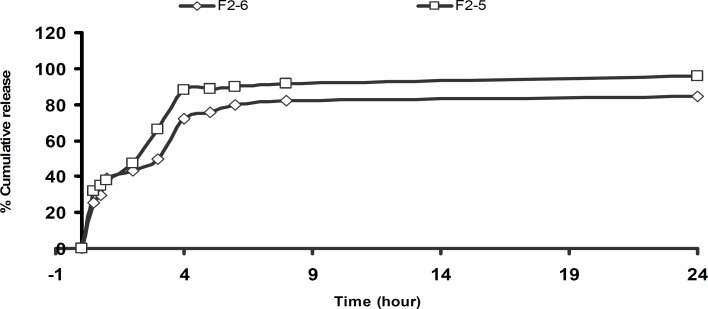
Cumulative percent release of theophylline from microspheres F_2_ prepared with different dispersing medium concentration

**Figure 8:**
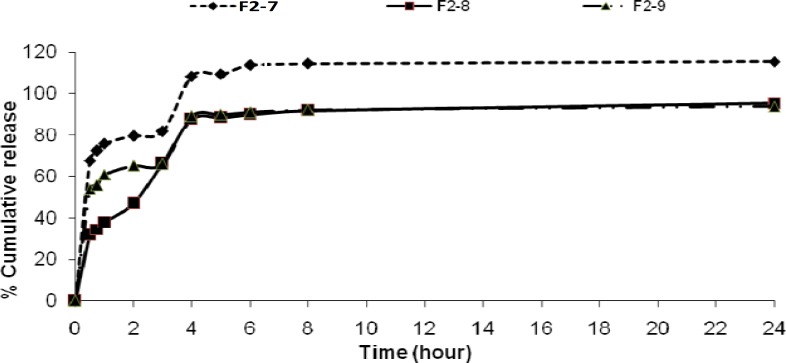
Cumulative percent release of theophylline from microspheres F_2_ prepared with different non-solvent amount

## Discussion

It is very important to carefully select the solvent combination and processing medium to enable the formation of double emulsion and solvent extraction and evaporation by a combination. TH is a water soluble drug and was dissolved in the water, forming the aqueous phase. Acetonitrile is a unique organic solvent which is polar, water miscible and oil immiscible. All other polar solvents like methanol, ethyl alcohol, ethylacetate, acetone, dimethylsulphoxide and tetrahydrofuran are oil-miscible and do not form emulsions of the polymer solution in oil (4, 20). Dichloromethane is non-polar and oil miscible. Using acetonitrile alone as a solvent did not ensure formation of a stable emulsion, and non-polar solvent such as dichloromethane was included to decrease polarity of the acetonitrile solution (4, 19). Ethylcellulose has been reported as one of the important polymeric materials used in microcapsules and microencapsulation formulations (14). This is due to its high safety, good stability, easy fabrication and cheapness (14). The drug-polymer ratio was varied by maintaining the amounts of polymer and solvent constant in all preparations, and also by changing the amount of drug. The encapsulation efficiency of the drug depended on the solubility of the drug in the solvent and continuous phase. Similar observation has been reported by Youan (22). Using higher amounts of the drug caused a slight increase in viscosity of dispersed phase. Entrapment efficiency of polypeptides was increased by enhancing the viscosity builders (23). The reason for increased production yield at high drug polymer ratios could be due to increased diffusion rate of solvents (acetonitrile and dichloromethane 1:1) from concentrated solutions into initial emulsion. 

Size of microcapsules was found to be increased with an increase in the concentration of drug (Table 2). A volume-based size distribution of drug, polymer, and drug loaded microcapsules indicated a log–probability distribution. SEM studies revealed that the microcapsules were almost spherical in shape (24, 25). Generally, an increase in the ratio of drug to polymer would increase the viscosity of dispersed phase (18, 26, 27). This increase in the viscosity of internal phase should normally reduce the rate and may be the extent of solvent partitioning into the external phase which should then lead to slow solidification of particle, hence an increase in microsphere particle size. In fact viscosity of dispersed phase was increased from F_1_ (0.25:1) to F_4_ (1:1). When the dispersed phase with higher viscosity was poured into the dispersion medium, bigger droplets were formed with larger mean particle size.

Microcapsules were formed after a series of steps like solvent extraction and solvent evaporation and addition of non-solvent. The solvents of the system were removed by a combination of extraction and evaporation. Components of the MSS can be selected from any of the commonly available organic solvents such as dichloromethane, ethyl acetate, acetone, acetonitrile, ethanol, etc (4). Having chosen oil as the processing medium, it is imperative that the solvent for polymer be immiscible with oil. Acetonitrile is a unique organic solvent which is polar, water-miscible and oil-immiscible. All other polar organic solvents like methanol, ethyl alcohol, ethyl acetate, acetone, dimethylsulphoxide and tetrahydrofuran are oil-miscible and do not form emulsions of the polymer solution in oil (4). With oil as a processing medium, use of acetonitrile alone as a dispersed medium did not ensure formation of a stable emulsion, and a non-polar solvent such as dichloromethane was included to decrease polarity of the acetonitrile solution. In this study, the mixed solvent system comprising 1:1 proportions of acetonitrile and dichloromethane was used. Therefore, during the formation of microcapsules, dichloromethane was extracted by liquid paraffin and acetonitrile was evaporated during stirring. One method of ensuring high entrapment efficiency of water-soluble active ingredients is to use a hydrophobic processing medium out of which the hydrophobic macromolecule is unlikely to migrate. Acetonitrile-liquid paraffin combination has been used for small molecules (28), while acetonitrile-vegetable oil combination has been used for small proteins like insulin (29). Each step of microsphere preparation was keenly observed to understand the effect on the particle size, total entrapment and release profiles of the drug loaded microcapsules. 

Depending on therapeutic requirements, microspheres with varying drug contents could therefore be prepared through variation of the theoretical drug loading (2). Increasing the amount of drug (drug: polymer ratio) in the organic phase slightly increased the viscosity of the primary W/O emulsion. This possibly stabilized the internal aqueous phase against coalescence and hence polymer loss to the external phase. The SEM of TH microspheres (F_2_) obtained by emulsion technique is shown in Figure 1. It can be seen that microspheres has crystalline irregular particles on the smooth surfaces (Free drug) which can be spherically agglomerated and microencapsulated. The drug may have been dispersed in crystalline or amorphous form or dissolved in the polymeric matrix during formation of the microspheres (30).

According to Figure2, the intensity of peak for drug loaded microspheres was the same as that related to pure drug, indicating the crystalline nature of drug in microcapsules. The intensity of the drug peak is decreased as the drug-loading is decreased. This may be due to a decrease in the degree of TH crystallinity in ethylcellulose microcapsules at lower drug-loading, indicating a mixture of both crystalline and amorphous forms of drug in microcapsules. Dubernet *et al* reported the similar type of observations TH-loaded ethylcellulose microcapsules (31). Tamilvanan and Sa have reported the same kind of characters in indomethacin-loaded polystyrene microparticles (32). 

The X-ray diffraction profile of ethylcellulose polymer indicated the presence of a completely amorphous material; pure TH showed the classical diffractogram of the crystalline product. No major difference in the XRD patterns of the physical mixture and the drug loaded microcapsules was noticed. However, decreases in the peak intensity and the baseline shift of the diffractogram were observed in the case of the TH loaded microcapsules, when compared to that of the physical mixture. The XRD of TH and TH loaded microcapsules were compared and the peaks of TH loaded microcapsules showed lower intensity than the pure drug, as shown in Figure 3. This confirms the results obtained from DSC experiments.

As shown in Figure 4, the characteristic OH stretching, NH stretching, C-H stretching and C=O stretching of pure drug was unchanged in the spectra of the microcapsules. The results suggest the stability of the drug during the encapsulation process and revealed the absence of drug-polymer interaction.

Theophylline is water soluble with less affinity to distribute from internal phase of initial emulsion to oily phase (outer phase in second emulsion). Therefore decreased drug content was seen in comparison to the theoretical drug content. Table 2 also shows that the stirring rate employed had effect on particle size diameter. It has been reported that the stirring rate of emulsion at the time of manufacturing influences the particle size and, in some cases, the size distribution of the prepared microparticles (33). Increasing the mixing speed generally results in decreased microsphere mean size (34), as it produces smaller emulsion droplets through stronger shear forces and increased turbulence. The extent of size reduction that is attained depends on the viscosity of disperse and continuous phases, the interfacial tension between the two phases.

At stirrer speed of 1500 rpm (F_2_-3), the resulting high turbulence, caused frothing and adhesion to the container wall. The mean particle size of microcapsules decreased because high stirring rate could prevent droplet coalescence in the oil medium. The desired spherical and not aggregated microcapsules were obtained at stirring speeds of 1000 rpm (F_2_-2, Table 2). Any increase in mean particle size at lower stirring rate as 500 rpm (F_2_-1) can be attributed to increased tendency of globules to coalescence and aggregates. 

As the volume of processing medium was increased, the emulsion droplets probably moved freely in the medium, thus reducing collision induced aggregation and yielding small (55.24% to 38.17%) and uniform microcapsules. This could also be the reason for higher drug extraction into the processing medium resulting in lower entrapment efficiency. 

The mean particle size decreased with increasing amount of non-solvent (Table 2). This is probably a consequence of solidification of the oil droplets with n-hexane. Spherical microcapsules were formed when the n-hexane content was at 10 ml. The *n-*hexane, non-solvent for the polymer added at this stage may lead to a quick precipitation of the polymer leaving the surface of microcapsules porous (21). For all formulations, a first initial burst release occurred. It was demonstrated that it was due to the adsorption of TH onto the outer surface of the microcapsules. Moreover, the burst release could also be explained by the imperfect encapsulation of the drug inside microcapsules, resulting from the unstable nature of the emulsion droplets during the solvent removal step. This potential instability may cause a part of the loaded drug to relocate at the microsphere surface, thereby rapidly released (35). In most cases, a biphasic dissolution profile was observed at pH 7.4: the initial rapid drug leakage generally ended very early (within first 30-60 min after the change of dissolution medium pH to 7.4); for the remaining time, nearly linear behavior was observed. It can be supposed that the first portion of the curves is due to TH dissolution, which starts immediately after the beginning of the test for the portion of drug on the surface of microparticles. After such a phase, two phenomena can combine in enhancing in the diffusion of the remaining dispersed drug into the bulk phase as well as the formation of pores within the matrix due to the initial drug dissolution; particle wetting and swelling which enhances the permeability of the polymer to the drug (35) (Figure 5). The results indicated that factors such as polymer-drug ratio, stirring speed, volume of processing medium of secondary emulsification and non-solvent concentration in secondary emulsification govern the drug release from these microcapsules. Drug release rates increased with increasing amounts of TH in the formulation. Higher level of TH corresponding to lower level of the polymer in the formulation resulted in an increase in the drug release rate. As more drugs are released from the microcapsules, more channels are probably produced, contributing to faster drug release rates. Only formulations F_2_ was prolonged release, which could be due to the thicker polymer membrane that sustains the release rate. 

According to Table 3, the lowest DE was observed for theophylline SR^®^ (73.72%) and the nearest DE to the dissolution efficiency of the commercial tablet belonged to formulation F_1_ (80.48%) (*P*< 0.05). The value of t_50% _varies between 3 (F_2_ formulation) to 4 hrs (theophylline SR). The results of similarity factor (f_1_) showed that the release profile of microparticle formulations is dissimilar to the release profile of commercial tablet (Table 3). The results of release of microspheres were compared with release of theophylline SR^®^ tablet and microspheres release were high in pH 1.2 (more burst effect than SR tablet) and release rate of SR tablet in pH 7.4 was lower than microspheres (*P*< 0.05).

The change of stirring speed of the secondary emulsification process also influenced the drug release profile as shown in Figure 6. This may be attributed to the presence of greater amount of free drug on the surface of the microspheres with increasing the concentration of dispersing medium used for secondary emulsification process. There was no differences on the drug released from the microspheres at pH=1.2. The faster drug release was observed from microspheres which were prepared by the use of large volume of processing medium at pH =7.4 (Figure 7). This formulation also had less drug entrapment (F_2_-6). It may be due to the higher migration of drug to the surface of the microspheres during solvent evaporation from the freely moved emulsion droplets in large volume of processing medium. When the volume of the dispersing medium was increased a faster drug release was observed (Figure 8).

For studying the kinetics of drug release, dissolution data were fitted to different kinetics models including the zero-order, first order, Peppas and square root of time using linear regression (37, 38). The fit parameters to Higuchi, first-order, Peppas and zero-order equations are given in Table 3. The rate constants were calculated from the slope of the respective plots. High correlation was observed for the Peppas model. The data obtained were also put in Korsemeyer-Peppas model in order to find out *n* value, which describes the drug release mechanism. The ratio of M_t_/M_∞_ represents the fraction of drug released at time *t*. Square regression coefficient (R^2^) was calculated for each model (Table 3). The *n* value of microcapsules of different drug to polymer ratio was between 0.28-0.40, indicating that the mechanism of the drug release were diffusion controlled. 

## Conclusion

TH microcapsules were prepared using double emulsion (w/o_1_/o_2_) solvent diffusion method. Drug: polymer ratio, stirring speed, emulsifier and dispersing medium influenced the drug entrapped, loading efficiency, particle size and drug release of the microcapsules. The entrapment efficiency was almost high for all formulations. The best drug to polymer ratio was 0.5:1 (F_2 _formulation). The drug loaded microspheres (F_2_) showed 29.53% of entrapment, loading efficiency 88.59%, and mean particle size 757.01 µm. SEM studies showed that the microspheres were spherical. The encapsulation efficiency of selected formulation (F_2)_ was influenced by changing the stirring speed of the second emulsification process, non-solvent concentration and dispersing medium concentration.

It was suggested that mechanism of drug release from microcapsules was diffusion controlled. Sustained release with initial burst release (loading dose) achieved with these formulations may reduce dose frequency and side effects as well as improved patient compliance.
